# Spatial phenotypic and genetic structure of threespine stickleback (*Gasterosteus aculeatus*) in a heterogeneous natural system, Lake Mývatn, Iceland

**DOI:** 10.1002/ece3.712

**Published:** 2013-08-06

**Authors:** Antoine Millet, Bjarni K Kristjánsson, Árni Einarsson, Katja Räsänen

**Affiliations:** 1 Department of Aquaculture and Fish Biology, Hólar University CollegeIS-551, Sauðárkrókur, Iceland; 2 Mývatn Research StationIS-660, Mývatn, Iceland; 3 Institute of Life and Environmental Science, University of IcelandIS-101, Reykjavík, Iceland; 4 Department of Aquatic Ecology, EAWAG and Institute of Integrative Biology, ETH ZurichÜberlandstrasse 133, CH-8600, Dübendorf, Switzerland

**Keywords:** Defense traits, feeding morphology, genetic divergence, microsatellite, phenotypic divergence

## Abstract

Eco-evolutionary responses of natural populations to spatial environmental variation strongly depend on the relative strength of environmental differences/natural selection and dispersal/gene flow. In absence of geographic barriers, as often is the case in lake ecosystems, gene flow is expected to constrain adaptive divergence between environments – favoring phenotypic plasticity or high trait variability. However, if divergent natural selection is sufficiently strong, adaptive divergence can occur in face of gene flow. The extent of divergence is most often studied between two contrasting environments, whereas potential for multimodal divergence is little explored. We investigated phenotypic (body size, defensive structures, and feeding morphology) and genetic (microsatellites) structure in threespine stickleback (*Gasterosteus aculeatus*) across five habitat types and two basins (North and South) within the geologically young and highly heterogeneous Lake Mývatn, North East Iceland. We found that (1) North basin stickleback were, on average, larger and had relatively longer spines than South basin stickleback, whereas (2) feeding morphology (gill raker number and gill raker gap width) differed among three of five habitat types, and (3) there was only subtle genetic differentiation across the lake. Overall, our results indicate predator and prey mediated phenotypic divergence across multiple habitats in the lake, in face of gene flow.

## Introduction

The balance between divergent natural selection and constraining gene flow is central for attempts to understand diversification of natural populations (reviewed in Lenormand 2002; Garant et al. 2007; Crispo 2008; Räsänen and Hendry 2008). Spatial abiotic (e.g., temperature, structural complexity) and biotic (e.g., prey and predator types) environmental variation can lead to divergent natural selection and promote adaptive divergence and ecological speciation (Schluter 2000). However, when geographic barriers are absent or environmental differences are minor, gene flow may constrain divergence (e.g., Lenormand 2002; Garant et al. 2007; Crispo 2008; Räsänen and Hendry 2008) and facilitate phenotypic plasticity or increase trait variance over local genetic differentiation (e.g., Hedrick et al. 1976; Sultan and Spencer 2002). Apart from few studies along environmental gradients (e.g., Antonovics 2006), most studies on selection versus gene flow balance have compared phenotypic and genetic divergence between two contrasting environments (e.g., Schneider et al. 1999; Langerhans et al. 2003; Nosil and Crespi 2004; Siwertsson et al. 2010). In contrast, relatively few studies have studied divergence across multiple habitat types (but see Ólafsdóttir et al. 2007a; Ólafsdóttir and Snorrason 2009) or across habitats with known extensive temporal variation. Yet such fine spatial dynamics can be of great importance as promoters or constraints of diversification (e.g., Levene 1953; Scheiner and Holt 2012).

Intralacustrine freshwater fish provide an interesting opportunity to study spatial variation in the extent of phenotypic and genetic divergence across multiple habitats. First, adaptive divergence and ecological speciation have been repeatedly documented in a range of empirical systems (e.g., Arctic charr *Salvelinus alpinus*, Snorrason and Skúlason 2004; Klemetsen 2010; threespine stickleback *Gasterosteus aculeatus*, McKinnon and Rundle 2002; Hendry et al. 2009; Eurasian perch *Perca fluviatilis* and roach *Rutilus rutilus*, Svanbäck et al. 2008; and whitefish *Coregonus* sp., Douglas et al. 1999; Hudson et al. 2011). The ecological drivers of divergence are typically linked to resource availability and competition (e.g., Smith and Skúlason 1996; Hendry et al. 2002, 2009; Svanbäck et al. 2008; Bolnick et al. 2010). Second, although lakes often encompass substantial spatial heterogeneity, such as variation in benthic habitats and along depth gradients (e.g., Dodds and Whiles 2010), they often lack geographic barriers to dispersal, providing ample opportunities for interactions between divergent natural selection and gene flow.

Studies on natural populations at early stages of divergence are particularly enlightening as they allow inferences on relative role of interacting ecological and evolutionary processes (e.g., Pelletier et al. 2009 and references therein). Northern freshwater systems, such as Icelandic lakes are young – having been available for colonization since the end of the last glacial period (ca. 10,000–14,000 years ago; Skúlason et al. 1999) – and commonly show high resource availability relative to the number of species present (Smith and Skúlason 1996). Here, we focus on threespine stickleback inhabiting the highly spatially and temporally variable Lake Mývatn, Iceland (Einarsson and Gulati 2004).

Stickleback show high propensity for colonization of, and adaptation to, different aquatic environments. Colonization of freshwater by marine stickleback has given rise to repeated parallel divergence and ecological speciation (Bell and Foster 1994; McKinnon and Rundle 2002; Hendry et al. 2009), in particular between anadromous–freshwater resident, benthic–limnetic, lake–stream, and mud–lava environments. The traits that typically undergo divergence in these systems include body size and shape, predator defense and feeding morphology (reviewed in Bell and Foster 1994; McKinnon and Rundle 2002; Hendry et al. 2009). Moreover, a recent study on stickleback in Lake Thingvallavatn, Iceland, found complex patterns of divergence across lava, mud, and Nitella habitats (Ólafsdóttir and Snorrason 2009), but most other studies on stickleback divergence have focused on two habitat types (e.g., benthic vs. limnetic). Potential for intralacustrine divergence across multiple habitat types has hence been largely unexplored in stickleback.

In Lake Mývatn, two morphs, mud and lava, of stickleback have previously been described (Kristjánsson et al. 2002; Ólafsdóttir et al. 2007b). The Mývatn system is particularly interesting for studies on multimodal spatial divergence because (i) it consists of several habitat types – putatively promoting divergent selection; (ii) the two main basins of the lake differ in both habitat types and stickleback population densities (Table S1) – potentially promoting or constraining divergence between the basins; and (iii) long-term monitoring (over 30 years) shows strong temporal dynamics in stickleback and chironomid midge (stickleback prey) population densities (Einarsson and Gulati 2004) – potentially resulting in spatiotemporal variation in selection. Importantly for our goals here: (1) there is spatial variation across the lake in vegetation and temperature (Table S1), abundance of piscivorous predators (Guðbergsson 2000; Einarsson and Gulati 2004), as well as midge and zooplankton communities (Einarsson and Gulati 2004) – indicating potential for multimodal divergent selection on stickleback; and (2) there are no physical barriers to dispersal, nor apparent strongly unsuitable areas among the different habitats or between the two basins – indicating high potential for gene flow.

We examined phenotypic divergence in body size, defense, and feeding morphology – traits typically under selection in stickleback, and used microsatellite markers to quantify the extent of genetic structure (a proxy for gene flow). We tested for divergence at two spatial scales: across five habitat types and between the two basins. We made the following main predictions: (1) if environmental differences promote genetic or plastic divergence, we expect phenotypic differences among the different habitat types and/or basins; (2) if gene flow constrains adaptive divergence or divergent selection is weak, we expect to see low phenotypic divergence across environments; and (3) if divergent selection imposes strong constrain on gene flow, we expect to see reduced gene flow across the different habitat types/between the basins. The work presented here is intended to establish the extent of multimodal spatial divergence during the breeding season (a standard approach in stickleback divergence studies) and is the first step in our exploration of diversification in a spatiotemporally heterogeneous system.

## Material and Methods

### Study system

**Figure 1:**
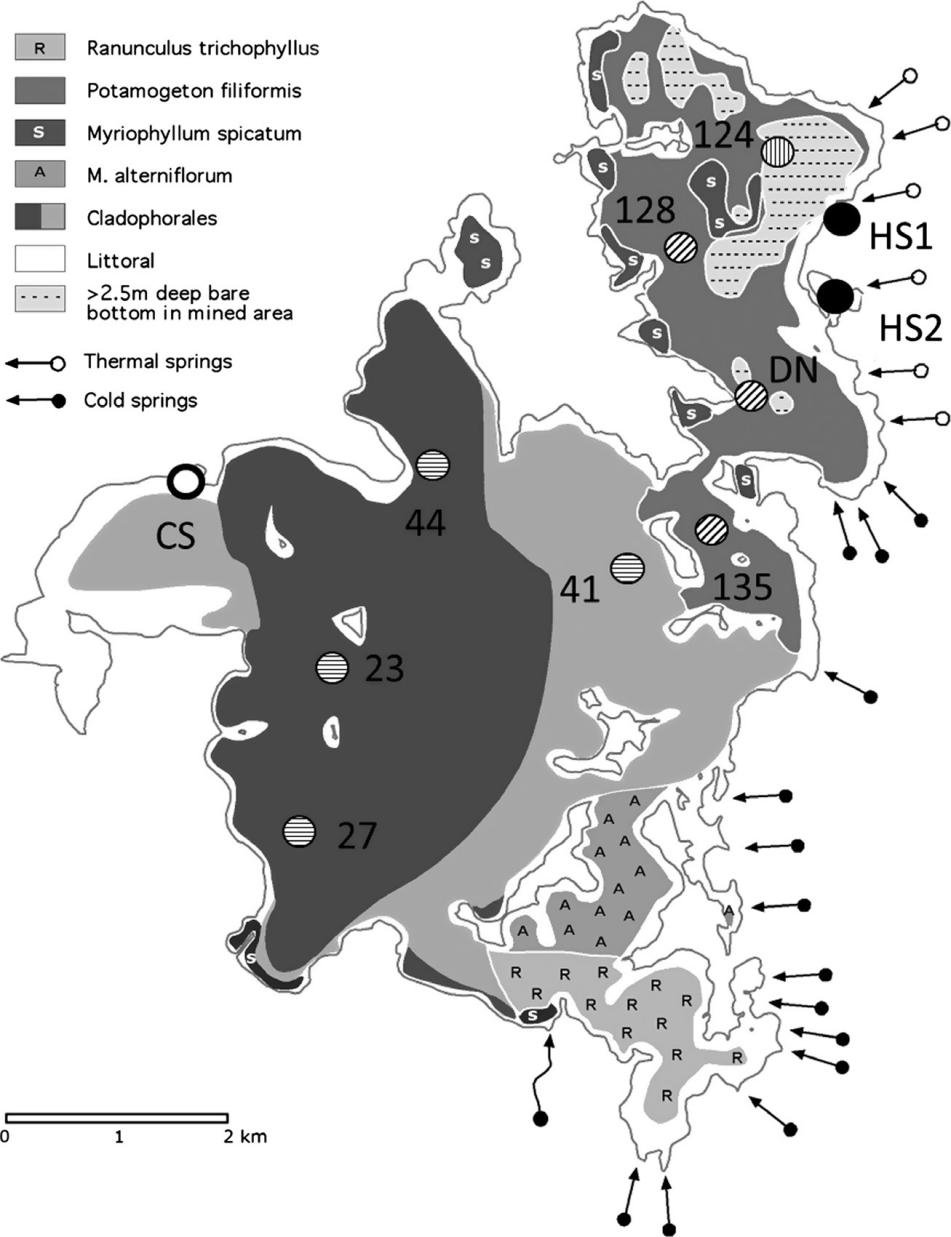
Map of Lake Mývatn, North East Iceland, showing the dominating benthic macrophytes, the main springwater inflows, and the 11 sampling sites of threespine sticklebacks. The two shades of gray used for Cladophorales reflect its relative density (lighter: less dense, darker: more dense). Sampling sites are marked as circle. Black filled: Warm habitat; vertical striped: Mined habitat; diagonal striped: Pondweed habitat; horizontal striped: Cladophorales habitat; and black circle: Shore habitat. Edited from Einarsson et al. (2004).

Lake Mývatn (65°36'N, 17°00'W; 37 km^2^) is a shallow eutrophic lake (Fig. 1, Table S1), which was formed about 2300 years (ca. 2300 stickleback generations) ago following a volcanic eruption (Einarsson and Gulati 2004). The North (N) basin is smaller (8.5 km^2^), deeper (up to 5.5 m) due to mining operations from 1967 to 2004 (Einarsson and Gulati 2004), and is mainly fed by warm water springs (up to 30°C, Ólafsson 1979) on its East shore (Fig. 1). The South (S) basin is larger (28.2 km^2^), shallower (max. depth 4 m), and fed by cold water springs (ca. 5°C; Ólafsson 1979) on the E shore. The two basins also differ in biotic characteristics, such as vegetation type (Fig. 1), phytoplankton, zooplankton and chironomid midge densities and community composition, as well as stickleback and bird densities (Dickman et al. 1993; Einarsson and Gulati 2004).

To facilitate predictions about environmental drivers of diversification, we divided the lake into five different habitat types based on vegetation/substrate cover, Cladocera communities and water temperature (Fig. 1, Table S1). The “Warm” habitat (N basin) is characterized by a warm water inflow, lava rocks, silica mud substrate with sparse pondweed (*Potamogeton filiformis*). The “Mined” habitat (N basin) corresponds to the deepest part of the lake with very muddy substrate, no vegetation, and partly anaerobic conditions due to the previous mining activities. The “Pondweed” habitat corresponds to shallow areas (most of N basin and North end of S basin) with pondweed (*Potamogeton* spp.) as the main vegetation. The “Cladophorales” habitat (S basin) corresponds to a large area with bare mud interrupted by patches of mat-forming Cladophorales algae. The “Shore” habitat (S basin) corresponds to a ca. 1 m deep shoreline with sparse rocks and vegetation. The Warm habitat is consistently warm (20–23°C, Table S1), whereas all other habitats are on average much colder and follow the ambient temperature (average range during summer 11–13°C: Table S1).

### Stickleback sampling

We sampled stickleback from 11 sites across the lake (Fig. 1, Table S1) based on sampling stations used in the long-term monitoring of the lake (e.g., Gudmundsson 1996; Einarsson and Gulati 2004). The sites were classified into (A) basin and (B) habitat types (Fig. 1, Table S1). The previous description of mud and lava stickleback (Kristjánsson et al. 2002; Ólafsdóttir et al. 2007b) was based on samples from one site in the S basin (site 135, here “Pondweed”; Fig. 1) and one site in the N basin (HS2, here “Warm”; Fig. 1). This work showed that mud fish tend to be larger and have relatively larger heads, longer gill rakers, and longer spines than lava fish (Kristjánsson et al. 2002), and mud males have relatively smaller brains than lava males (Kotrschal et al. 2012). Both microsatellite and mtDNA markers indicated that mud and lava stickleback are genetically distinct (Ólafsdóttir et al. 2007b).

Stickleback were sampled during the breeding season in 2009 in conjunction with the annual long-term monitoring. On 20–24 June, five unbaited minnow traps (Dynamic Aqua-Supply Ltd, Surrey, BC, Canada, mesh size 3.2 mm) per site were set out overnight for approximately 12 hours/site. All sampled stickleback were transferred to the Mývatn research station where they were frozen for later processing.

As local population densities may influence intensity of resource competition, as well as potential for gene flow, all fish in the five traps/site were counted and total catch used as an estimate of local population density (Table S1). Stickleback were subsequently sorted based on their total length to Small (<50 mm; i.e., 0+ stickleback) and Large (≥50 mm; which represent all +1 age classes; Gíslason et al. 1998). For each habitat, 27–534 fish were kept for body size analyses (total *N* = 1139).

Up to ca. 50 Large stickleback/site (henceforth: subsample) were randomly selected for further phenotypic and genetic analysis. When less than 50 Large individuals/site were available, Small stickleback >35 mm in size were added to reach ca. 50 individuals/site. The right pectoral fin from each of the subsampled fish was clipped and preserved in 95% ethanol for genetic analyses. Fish were subsequently preserved in 5% buffered formalin for a minimum of 3 weeks, and thereafter rinsed with water and stored in 70% ethanol. Fish ≤35 mm where preserved whole in 95% ethanol and were not used in this study except for calculation of total catch.

### Phenotypic variation

For all fish, body size was measured as a general indicator of life-history variation. For the subsample, predator defense (lateral plate number, length of the first two dorsal spines, and the left pelvic spine) and feeding morphology (gill raker number [GRN], gill raker length [GRL], and gap width [GW]) was measured as indicators of functionally important phenotypic variation. In freshwater stickleback, plate number may decrease in response to reduced risk of predation from birds, while spine length may increase in response to selection by gape-limited predators (e.g., Reimchen 1994) or decrease in populations inhabiting lava habitats (Kristjánsson et al. 2002; Ólafsdóttir et al. 2007a; Ólafsdóttir and Snorrason 2009). Longer and more gill rakers and narrower gaps between gill rakers increase feeding efficiency on small prey (e.g., limnetic zooplankton), whereas shorter and fewer gill rakers and wider gaps are better suited for feeding on large prey items (e.g., benthic macroinvertebrates; e.g., Schluter and McPhail 1992; Bolnick 2004). Body size, GRL, and GW are known to be somewhat plastic (e.g., Day et al. 1994; Berner et al. 2008), whereas variation in plate number, spine length, and GRN often have a stronger genetic basis (Peichel et al. 2001; Colosimo et al. 2004; Shapiro et al. 2004).

For body size, total length of each individual was measured (to the nearest 1 mm) using a ruler. All fish in the subsample were sexed by visual examination of the gonads and noted as male, female, or immature. Fish were bleached (1:1 ratio of 3% H_2_O_2_ and 1% KOH) and stained (solution of alizarin red in 1% KOH) (Bell 1982) to aid counting of meristic characters and measuring of morphological features.

The number of lateral armor plates on the left side of the fish, and length of spines (the 1st and 2nd dorsal spine and the left pelvic spine, SP1, SP2, and PSP, respectively), were measured under a stereomicroscope (Leica MZ12, Wetzlar, Germany) fitted with an ocular micrometer. For quantification of feeding morphology, the first gill arch was removed from the left side of the fish, wetted in 70% ethanol, mounted between two glass plates, and photographed using a digital camera (Nikon Coolpix 4500; 4 Mpixels, Nikon, Tokyo, Japan) mounted onto a stereomicroscope (Leica MZ12). To quantify feeding morphology, a) the total number of long gill rakers (incl. both long and shorter arch) (GRN), b) the length (mm) of the first four gill rakers on the long arch (GRL), and c) the width of the gap (mm) between gill rakers 1–4 (GW) on the long arch was measured. For GRL and GW, averages across the four rakers or three gaps, respectively, were used in the analyses. All measurements were done (to the nearest 0.01 mm) from digital images using the public domain program ImageJ v1.43u (http://rsbweb.nih.gov/ij/; Schneider et al. 2012). Straight lines were used to measure GW and curved lines to measure GRL. In this study, 397 (36–145 per habitat and 77–220 per sex) and 343 (28–127 per habitat and 66–202 per sex) individuals were measured for the defensive and feeding traits, respectively.

### Genetic variation

Two hundred and sixty-seven stickleback (30–92 per habitat) were used for population genetic analyses (Table 1). DNA was extracted from fin clips using a standard proteinase K lysis followed by a salt-out purification (Aljanabi and Martinez 1997). To assess genetic structure, 16 microsatellite markers (Gac1097, Gac1125, Gac2111, Gac4170, Gac5196, Gac7033, Stn26, Stn30, Stn70, Stn96, Stn130, Stn173, Stn174, Stn185, Stn196 and Gaest66F; Largiadèr et al. 1999; Peichel et al. 2001; Mäkinen et al. 2008) were initially used. Seven of these loci (Gac1097, Gac1125, Gac2111, Gac4170, Gac5196, Gac7033, and Stn26) have been used in previous analyses of Icelandic stickleback (Ólafsdóttir et al. 2007a,b; Ólafsdóttir and Snorrason 2009). Gac1125, Gac2111, Gac7033, Stn26, Stn96, and Stn130 have been identified as putative QTLs (quantitative trait loci) in other stickleback studies (Gac2111, Gac7033, Stn26, Stn96: spine length; Gac1125: plate width; Stn130: spine length and number of short gill rakers; Peichel et al. 2001; Colosimo et al. 2004; Ólafsdóttir et al. 2007a; Mäkinen et al. 2008).

**Table 1:** Population genetic variation of threespine stickleback in five habitat types of Lake Mývatn for the 12 microsatellite markers

	Warm (*N* = 60–68)				Mined (*N* = 30)				Pondweed (*N* = 84–90)				Cladophorales (*N* = 32–35)				Shore (*N* = 36–39)			
					
	AR	*H*_E_	*H*_O_	HWE	AR	*H*_E_	*H*_O_	HWE	AR	*H*_E_	*H*_O_	HWE	AR	*H*_E_	*H*_O_	HWE	AR	*H*_E_	*H*_O_	HWE
Average	6.1	0.53	0.52		5.8	0.53	0.50		6.0	0.56	0.55		6.0	0.54	0.49		5.8	0.54	0.54	
Gac1097	7.1	0.70	0.71	0.79	8.0	0.70	0.67	0.35	7.3	0.70	0.66	0.34	8.7	0.73	0.61	0.20	7.7	0.75	0.80	0.57
Gac5196	6.1	0.72	0.75	0.66	5.0	0.65	0.63	0.42	6.3	0.71	0.79	0.33	7.6	0.75	0.73	0.52	4.8	0.70	0.77	0.53
Gac4170	8.8	0.78	0.81	0.41	10.0	0.81	0.77	0.42	8.7	0.80	0.84	0.25	7.9	0.81	0.78	0.09	6.5	0.72	0.67	0.71
Stn30	6.7	0.62	0.52	0.20	5.0	0.64	0.53	0.15	6.1	0.66	0.62	0.62	5.7	0.62	0.66	0.86	4.7	0.58	0.61	0.74
Stn173	3.0	0.29	0.30	1.00	4.0	0.30	0.23	0.02	3.4	0.32	0.32	0.46	3.0	0.39	0.31	0.34	2.8	0.25	0.25	0.19
Stn196	4.3	0.28	0.28	0.28	3.0	0.26	0.30	1.00	4.0	0.35	0.35	0.86	2.9	0.31	0.26	0.38	4.6	0.40	0.39	0.87
Stn174	4.9	0.54	0.58	0.40	3.0	0.52	0.50	1.00	2.7	0.51	0.51	1.00	2.0	0.51	0.40	0.31	3.7	0.52	0.42	0.26
Gac1125	11.1	0.68	0.71	0.54	11.0	0.72	0.73	0.25	11.5	0.75	0.79	0.64	14.4	0.72	0.64	0.39	13.0	0.75	0.76	0.82
Gac2111	5.2	0.37	0.37	0.69	6.0	0.45	0.37	0.05	5.4	0.44	0.37	0.01	4.7	0.25	0.21	0.47	4.9	0.41	0.41	0.60
Gac7033	3.8	0.13	0.11	0.15	3.0	0.07	0.07	1.00	4.2	0.20	0.16	0.02	2.8	0.06	0.06	1.00	4.3	0.17	0.13	0.25
Stn130	8.5	0.77	0.70	0.43	8.0	0.77	0.70	0.02	8.3	0.72	0.70	0.11	6.8	0.72	0.63	0.43	7.7	0.77	0.78	0.26
Stn26	3.5	0.43	0.43	0.54	4.0	0.44	0.47	1.00	4.0	0.60	0.54	0.72	4.9	0.62	0.57	0.32	4.8	0.49	0.50	0.77

AR, allelic richness; *H*_E,_ expected heterozygosity; *H*_O,_ observed heterozygosity; HWE, test of Hardy–Weinberg equilibrium (*P*-value).

Two multiplexed polymerase chain reactions (multiplex PCR) were used to amplify the loci. The first multiplex reaction consisted of Gac1097, Gac1125, Gac2111, Gac4170, Gac5196, and Gac7033 and was amplified following the protocol of the first multiplex used by Raeymaekers et al. (2007). The second multiplex consisted of the 10 remaining loci and was amplified according to the following protocol (J. A. M. Raeymaekers, pers. comm.): an initial activation step at 95°C for 15 min followed by 26 cycles of 30 sec at 95°C, 90 sec at 53°C, and 60 sec at 72°C. A final elongation step of 30 min at 60°C was then performed. PCR products were analyzed on a 3730 × L DNA Analyser (Applied Biosystems, Carlsbad, CA) and the GeneScan 500-LIZ was used as size standard. Genotypes were manually scored using Peak Scanner v1.0 (Applied Biosystems). As Stn96 and Stn185 did not give reliable scores, they were excluded from the analysis, which retained 14 microsatellites at this point (nine neutral and five putative QTLs).

### Statistical analyses

All continuous phenotypic traits (body size, GRL, GW, SP1, SP2, and PSP) were log transformed prior to statistical analysis. As GRL, GW, and spine lengths were highly correlated with body size (all Pearson *r* > 0.79, *P <* 0.001), analyses were conducted on residuals from linear regression of a given phenotypic measurement against the total length of each individual (Reist 1986).

Statistical analyses of phenotypic variation were run in two steps. The first set of models tested for differences among habitats (Warm, Mined, Pondweed, Cladophorales and Shore), and the second set of models for differences between the two basins (N vs. S). Body size in the large sample (all individuals above 40 mm) was analyzed using analysis of variance (ANOVA). We did not test for the effect of sex on body size in this data set as gonads were not inspected for most of the individuals (due to logistic reasons) and external visual inspection would render sex determination potentially unreliable. The ANOVAs on body size models included only habitat (or basin) as fixed factor. ANOVAs were followed by visual inspection of body size frequency distributions to allow inferences on age structure (Gíslason et al. 1998).

Defense and feeding morphology were analyzed separately using two sets of type II multivariate analysis of variances (MANOVAs) including either (1) all defense traits or (2) all feeding morphology traits. The first set of models included Habitat (or Basin), Sex (male, female, or immature) and the Habitat (or Basin) × Sex interaction as fixed factors. In cases where the interaction was not significant, it was removed from the analysis and only results from the final models are presented here. Post hoc analyses of relevant pairwise differences were subsequently performed using post hoc Tukey tests. Before analyses, assumptions of (M)ANOVAs were confirmed with QQ plots. All statistical analysis of phenotypic traits were performed using R (v. 2.15.1; R Development Core Team 2011). (M)ANOVAs were performed using the package “car” (Fox and Weisberg 2011) and post hoc analyses were performed using the package “agricolae” (de Mendiburu 2012).

### Genetic analyses

Departures from the Hardy–Weinberg equilibrium (HWE) were calculated using the HWE exact test implement in GENEPOP v4.1.0 (Raymond and Rousset 1995). Locus Gaest66F was excluded as only 14 individuals were heterozygote for this marker and locus Stn70 was also excluded as it did not support HWE (*χ*^*2*^_16_ = ∞, *P* < 0.0001; inclusion or exclusion of Stn70 did not alter the results, however. Results not shown). Hence 12 markers remained in further analyses. Genetic diversity was assessed using allelic richness (AR) and observed and expected heterozygosity (*H*_O_ and *H*_E_) using the package “Hierfstat” (Goudet 2005). Overall and pairwise *F*_ST_s were calculated using FSTAT v2.93 (Goudet 1995), and the overall and pairwise differentiation index *D* (Jost 2008) was calculated using the package “DEMEtics” (Gerlach et al. 2010). Genetic differentiation was assessed with the θ estimation of *F*_ST_ (Weir and Cockerham 1984) and *D* for all loci and across all sampling sites, as well as pairwise between habitats. 95% confidence intervals of *D*s were calculated with a 1000 iterations bootstrap implemented in “DEMEtics”. Isolation by distance (IBD) was calculated as an indicator of gene flow being reduced by geographic distance across all sampling sites (i.e., not per habitat) using Mantel test of pairwise *F*_ST_s against pairwise geographic distances between each pair of sites. All results were corrected for multiple comparisons using the B-Y method recommended by Narum (2006).

In order to assess whether our microsatellite loci behave neutrally, LOSITAN (Beaumont and Nichols 1996; Antao et al. 2008) was run including all 12 loci using an infinite allele model and 100,000 simulations. Although all loci behaved neutrally (see Results), loci specific pairwise *F*_ST_s and *D*s (between habitats) were calculated for markers Gac1125, Stn26, and Stn130 because (i) Gac1125 previously indicated high level of divergence in Mývatn stickleback (G. Á. Ólafsdóttir, pers. comm.), and (ii) Stn26 and Stn130 have been associated with divergence in spine length and GRN, respectively, in Scandinavian and Belgian stickleback populations (Raeymaekers et al. 2007; Mäkinen et al. 2008).

## Results

### Population density

Total catch was larger in all three N basin habitats (Warm range: 650–1952; Mined: 1836 and Pondweed: 64–1624) than in the two S basin habitats (Cladophorales: 32–300 and Shore: 65), indicating that population densities are over 10-fold higher in the N (mean ± SD: 1511.4 ± 513.4) than in the S basin (107.3 ± 103.1, Table S1).

### Phenotypic variation

#### Body size

Body size ranged from 40 to 81 mm across the lake (grand mean ± SE: 54.8 ± 0.30 mm; Table 2) and differed significantly among habitats (*F*_4,1134_ = 10.72, *P* < 0.001) and between basins (*F*_1,1137_ = 45.58, *P* < 0.001). Stickleback in all N basin habitats (Warm: 53.6 ± 1.01 mm; Mined: 54.1 ± 1.01 mm and Pondweed: 55.0 ± 1.01 mm) were larger than stickleback in both S basin habitats (Cladophorales: 48.9 ± 1.02 mm and Shore: 47.9 ± 1.03 mm; all *P* < 0.02). Visual inspection of size frequency distribution (Fig. S1) further indicated that at least two adult size classes are abundant in the N basin, whereas the relative frequency of >55 mm fish was much smaller in the S basin (Gíslason et al. 1998).

**Table 2:** Mean (SD) and range of phenotypic traits measured in the threespine stickleback from Lake Mývatn

	Body size (mm)		GRN		GRL (residuals)		GW (residuals)	
				
	Mean (SD)	Range	Mean (SD)	Range	Mean (SD)	Range	Mean (SD)	Range
Basin								
North	54.6 (10.0)	40–81	20.1 (1.3)	16–23	−0.0059 (0.0172)	−0.0675–0.0409	−0.0014 (0.0061)	−0.0170–0.0190
South	48.7 (7.6)	40–76	19.8 (1.3)	16–23	−0.0075 (0.0155)	−0.0456–0.0549	−0.0013 (0.0045)	−0.0145–0.0104
Habitat								
Warm	53.6 (7.5)	40–72	20.3 (1.2)	18–23	−0.0082 (0.0168)	−0.0675–0.0284	−0.0034 (0.0051)	−0.0167–0.0070
Mined	54.1 (9.8)	40–79	20.1 (1.3)	16–23	−0.0015 (0.0158)	−0.0392–0.0269	0.0024 (0.0068)	−0.0127–0.0158
Pondweed	55.0 (10.7)	40–81	19.9 (1.3)	16–22	−0.0066 (0.0171)	−0.0487–0.0409	−0.0014 (0.0054)	−0.0170–0.0190
Cladophorales	48.9 (6.9)	40–70	19.9 (1.4)	17–23	−0.0084 (0.01662)	−0.0360–0.0549	−0.0010 (0.0046)	−0.0126–0.0074
Shore	47.9 (7.4)	40–76	19.6 (1.5)	16–22	−0.0046 (0.0144)	−0.0279–0.0276	−0.0015 (0.0040)	−0.0077–0.0104
Sex								
Female			19.9 (1.3)	16–23	−0.0112 (0.0147)	−0.0487–0.0276	−0.0019 (0.0061)	−0.0170–0.0190
Male			20.2 (1.2)	18–23	0.0050 (0.0176)	−0.0360–0.0549	−0.0010 (0.0047)	−0.0097–0.0136
Immature			19.8 (1.4)	16–23	−0.0113 (0.0151)	−0.0675–0.0215	−0.0001 (0.0045)	−0.0129–0.0074

	SP1 (residuals)		SP2 (residuals)		PSP (residuals)		Armor plates	
				
	Mean (SD)	Range	Mean (SD)	Range	Mean (SD)	Range	Mean (SD)	Range
Basin								
North	0.0020 (0.0116)	−0.0356–0.0444	0.0020 (0.0122)	−0.0368–0.0456	0.0014 (0.0136)	−0.0570–0.0430	5.3 (1.2)	3–9
South	−0.0029 (0.0120)	−0.0345–0.0288	−0.0028 (0.0120)	−0.0304–0.0285	−0.0018 (0.0126)	−0.0399–0.0346	5.3 (1.0)	4–8
Habitat								
Warm	0.0005 (0.0108)	−0.0234–0.0232	−0.0003 (0.0115)	−0.0251–0.0264	−0.0019 (0.0145)	−0.0377–0.0300	5.5 (1.0)	4–8
Mined	0.0080 (0.0120)	−0.0186–0.0356	0.0099 (0.0115)	−0.0144–0.0456	0.0086 (0.0136)	−0.0199–0.0430	5.1 (1.1)	3–7
Pondweed	0.0047 (0.0116)	−0.0356–0.0444	0.0038 (0.0123)	−0.0368–0.0430	0.0043 (0.0127)	−0.0570–0.0322	5.2 (1.2)	3–9
Cladophorales	−0.0032 (0.0122)	−0.0345–0.0288	−0.0035 (0.0115)	−0.0304–0.0230	−0.0029 (0.0122)	−0.0399–0.0346	5.3 (1.0)	4–8
Shore	−0.0091 (0.0111)	−0.0260–0.0129	−0.0081 (0.0123)	−0.0249–0.0205	−0.0043 (0.0123)	−0.0250–0.0170	5.3 (1.0)	4–7
Sex								
Female	−0.0041 (0.0127)	−0.0356–0.0285	−0.0034 (0.0127)	−0.0368–0.0330	−0.0032 (0.0142)	−0.0570–0.0404	5.2 (1.2)	3–9
Male	−0.0012 (0.0116)	−0.0309–0.0444	−0.0018 (0.0111)	−0.0240–0.0430	0.0002 (0.0113)	−0.0355–0.0243	5.4 (1.0)	4–8
Immature	0.0059 (0.0101)	−0.0241–0.0356	0.0062 (0.0118)	−0.0248–0.0456	0.0053 (0.0122)	−0.0250–0.0430	5.3 (1.0)	4–8

GRN, gill raker number; GRL, gill raker length; GW, gill raker gap width; SP1, first dorsal spine; SP2, second dorsal spine; PSP, pelvic spine correspond to residual length corrected for individual body size (see Methods).

#### Defense structures

**Figure 2:**
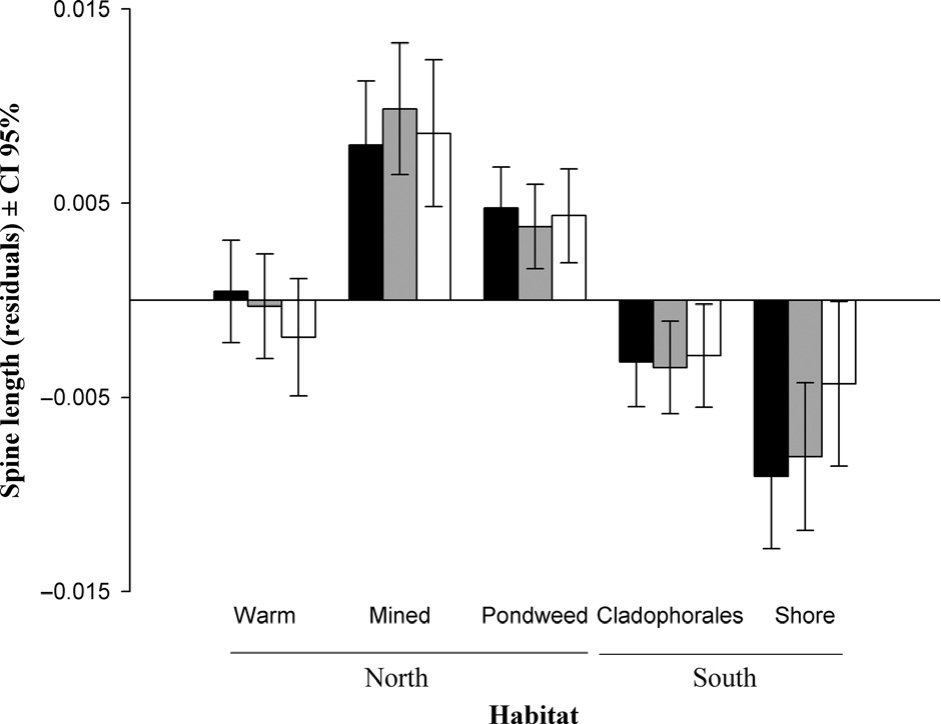
Mean ± CI 95% relative length of 1st dorsal spine (SP1; black bar); 2nd dorsal spine (SP2; gray bar); and pelvic spine (PSP; white bar) of stickleback from five main habitat types of Lake Mývatn. Data are residuals corrected for individual body size (see Methods).

Residual SP1 ranged from −0.0356 to 0.0444, residual SP2 from −0.0368 to 0.0456, and residual PSP from −0.0570 to 0.0430, and lateral plate numbers from 3 to 9 across all samples (Table 2). Defensive structures differed across habitats (MANOVAs: Wilks λ = 0.821, *F*_12,1026.8_ = 6.64, *P* < 0.001) and sexes (Wilks λ = 0.889, *F*_6,776.0_ = 7.82, *P* < 0.001), whereby lengths of all three spines differed among habitats (ANOVAs: *F*_4,390_ > 8.93, *P* < 0.001) and sexes (*F*_2,390_ > 11.57, *P* < 0.001), but plate number did not differ significantly across habitat types, basins, or sexes (Table 2; ANOVAs: all *P* > 0.25). Specifically, Mined stickleback had longer SP1s and SP2s (Fig. 2, Table 2) than did Cladophorales (Table 2; both *P* < 0.012) and Shore (Table 2; both *P* < 0.001) stickleback (Fig. 2), and Pondweed stickleback (Table 2) had longer SP1s and SP2s than Shore stickleback (*P* < 0.006, Fig. 2). Mined stickleback also had longer PSPs than Warm, Cladophorales, and Shore stickleback (Fig. 2, Table 2; all *P* < 0.035). Likewise, immature fish had relatively longer SP1s and SP2s (adjusted mean ± SE: SP1: 0.0059 ± 0.0012; SP2: 0.0062 ± 0.0012) than did either mature males (SP1: −0.0012 ± 0.0013; SP2: −0.0018 ± 0.0013) or females (SP1: −0.0041 ± 0.0009, SP2: −0.0034 ± 0.0009; all *P* < 0.021), and relatively longer PSPs (0.0053 ± 0.0014) than females (−0.0032 ± 0.0010; *P* = 0.046) but not males (0.0002 ± 0.0015; *P* = 0.18). Males and females did not differ in any spine length measurements (*P* > 0.54).

In the Basin analyses, there were significant differences between basins (MANOVAs Wilks λ = 0.893, *F*_3,391_ = 15.68, *P* < 0.001) and sexes (Wilks λ = 0.901, *F*_6,782_ = 6.99, *P* < 0.001), and these differences held for all three spine length measures (ANOVAs: all *P* < 0.001). In particular, N basin stickleback had relatively longer spines than S basin stickleback (Fig. 2, Table 2; *P* < 0.018).

#### Feeding morphology

**Figure 3:**
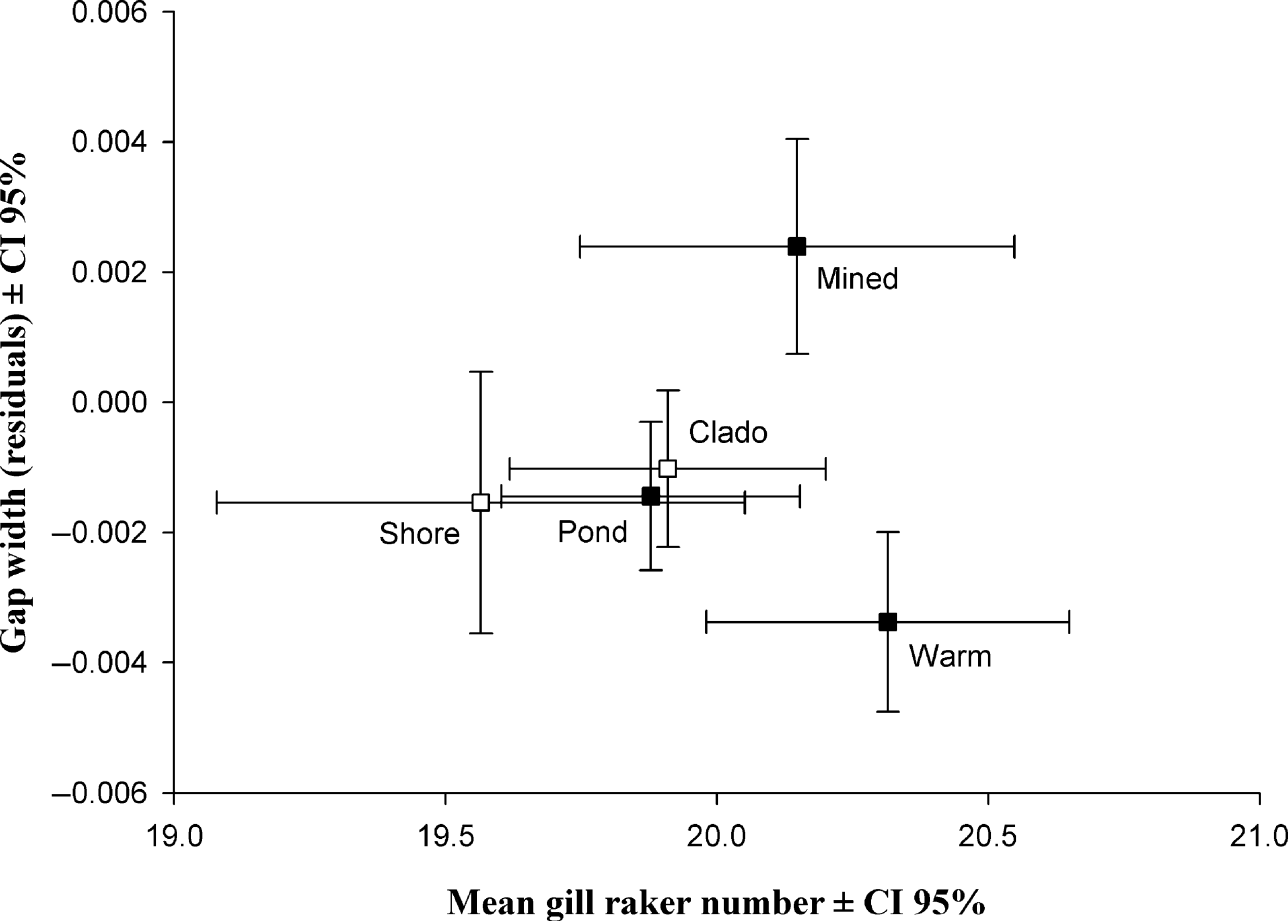
Mean ± CI 95% relative gill raker gap width as a function of gill raker number of stickleback sampled across five main habitat types of Lake Mývatn. Data on gap width are residuals corrected for individual body size (see Methods). Pond, Pondweed; Clado, Cladophorales habitat.

There were significant differences in gill raker morphology among habitats (MANOVA Wilks λ = 0.880, *F*_12,884_ = 3.66) and sexes (Wilks λ = 0.843, *F*_6,668_ = 9.93, both *P* < 0.001). These effects were primarily due to significant differences among habitats in relative GW (ANOVA *F*_4,336_ = 7.74, *P* < 0.001) and tendencially in GRN (*F*_4,336_ = 2.12, *P* = 0.078), and between sexes in relative GRL (*F*_2,336_ = 28.05, *P* < 0.001). Specifically, Mined stickleback (Table 2) had wider gaps than both Warm and Pondweed stickleback (Fig. 3, Table 2, both *P* < 0.001) and marginally wider gaps than Cladophorales stickleback (Fig. 3, Table 2, *P* = 0.062). Warm stickleback had narrower gaps than Cladophorales stickleback (Fig. 3; Table 2; *P* = 0.044) and tended to have more gill rakers than Shore stickleback (Fig. 3, Table 2, *P* = 0.061). Males had relatively longer gill rakers than both females and immature fish (Table 2; both *P* < 0.001), whereas there was no significant difference between females and immature fish in GRL (Table 2; *P* = 0.70). There were no significant differences in feeding morphology between the two basins (Wilks λ = 0.991, *F*_3,337_ = 0.98, *P* = 0.41).

### Genetic structure

Loci specific AR varied from low (2.0) to high (14.4) (Table 1). LOSITAN analysis suggested that all loci behaved neutrally (Fig. S2), and hence all 12 loci were pooled together in analyses of genetic structure. Overall habitat based neutral genetic structure was low and nonsignificant (*F*_ST_ = 0.004, *D* = 0.007; both *P* > 0.05), and there was no statistical evidence for IBD (Mantel's *r* = −0.019, *P* = 0.50). However, Mined stickleback differed significantly from Pondweed (*F*_ST_ = 0.009, *P* < 0.05; *D* = 0.019 [CI 95%: 0.011–0.032], *P* < 0.05), Cladophorales (*F*_ST_ = 0.007, *P* < 0.05; *D* = 0.017 [0.006–0.036], *P* > 0.05) and Shore stickleback (*F*_ST_ = 0.010, *P* < 0.05; *D* = 0.019 [0.008–0.034], *P* < 0.05; Table 3), indicating somewhat restricted gene flow between Mined and other habitats.

**Table 3:** Pairwise *F*_ST_s (upper) and pairwise Ds (lower) of microsatellite markers of stickleback sampled across five habitats in Lake Mývatn

	Neutral	Gac1125	Stn130	Stn26
Warm				
Mined	0.003	−0.002	−0.002	0.003
	0.007	−0.006	−0.004	0.002
Pondweed	0.005	0.004	−0.003	0.032
	0.007	0.009	−0.007	**0.036**
Cladophorales	0.005	0.003	0.000	0.036
	0.007	0.005	0.004	**0.039**
Shore	−0.001	−0.004	−0.005	−0.006
	−0.001	−0.012	−0.015	−0.006
Mined				
Pondweed	**0.009**	0.003	0.006	0.015
	**0.019**	0.003	0.017	0.019
Cladophorales	**0.007**	−0.001	−0.008	0.015
	0.017	0.000	−0.018	0.018
Shore	**0.010**	−0.007	−0.005	−0.008
	**0.019**	−0.021	−0.016	−0.008
Pondweed				
Cladophorales	0.001	**0.013**	0.007	−0.010
	0.005	**0.037**	0.020	−0.015
Shore	0.002	−0.004	0.009	0.012
	0.005	−0.013	0.026	0.016
Cladophorales				
Shore	0.003	0.001	−0.008	0.012
	0.006	0.004	−0.020	0.015

Bold values significantly different from zero (*P* < 0.05 after B-Y corrections). Neutral: all 12 loci pooled.

Pondweed and Cladophorales stickleback differed significantly in Gac1125 (*F*_ST_ = 0.013 and *D* = 0.035 [0.018–0.082], both *P* < 0.05; Table 3), while Warm stickleback differed significantly in Stn26 from both Pondweed (*F*_ST_ = 0.032; *P* > 0.05 and *D* = 0.036 [0.028–0.062], *P* < 0.02; Table 3) and Cladophorales stickleback (*F*_ST_ = 0.036; *P* > 0.05 and *D* = 0.039 [0.028–0.073], *P* < 0.02; Table 3). There was no significant differentiation in Stn130 among the habitats (Table 3).

## Discussion

We found subtle but significant phenotypic and genetic divergence of stickleback across Lake Mývatn, indicating multimodal divergence in absence of barriers to dispersal. In particular, stickleback differed in feeding morphology among three of the five main habitats, and in body size and spine length between the two ecologically distinct basins. Overall neutral genetic structure was low and nonsignificant, suggesting extensive gene flow across the lake. However, Mined stickleback differed significantly from Pondweed, Cladophorales, and Shore stickleback in neutral markers, and Warm stickleback from both Pondweed and Cladophorales stickleback in two loci (Gac1125 and Stn26) that are putative QTLs in European stickleback, providing some evidence for restricted gene flow across the lake.

### Phenotypic structure

Stickleback in the N basin (Warm, Mined, and Pondweed) were, on average, larger than stickleback in the S basin (Cladopohorales and Shore). Body size frequency distributions (Fig. S1) of adult stickleback further indicated that the relative frequency of >55 mm fish individuals was much higher in the N basin than in the S basin – likely reflecting differences in age structure of the adult populations (Gíslason et al. 1998). The exact reasons for the differences in body size/age structure between the N and S basin are unclear, but may indicate differences between the basins in life-history strategies (e.g., larger females are more fecund, Baker 1994) and/or plastic or genetic variation in responses to resource availability (e.g., Baker et al. 2011) and predation pressure (e.g., size selective predation; Reimchen 1994). Further studies on variation in body size and age structure in this system are clearly important as body size readily responds to environmental changes and natural selection in stickleback (e.g., Bell and Foster 1994; McKinnon and Rundle 2002; Hendry et al. 2009; Baker et al. 2011), is intimately linked to population density and population dynamics (e.g., Herrel et al. 2008; Rouyer et al. 2012), and age structure has implications for evolutionary responses (e.g., Charlesworth 1994; Engen et al. 2011).

We found that N basin stickleback had longer spines than S basin stickleback, which likely reflect differences in densities of gape-limited predators (e.g., Reimchen 1994). In Lake Mývatn, potential gape-limited predators include piscivorous diving birds (in particular, the horned grebe, *Podiceps auritus*, and the red-breasted merganser, *Mergus serrator*) and fish (Arctic charr, *S. alpinus* and brown trout, *Salmo trutta*). Densities of these main predators are two- to 10-fold higher in the N basin than in the S basin (Guðbergsson 2000; Einarsson and Gulati 2004; Á. Einarsson, unpubl. data, 1975–2012). Predation pressure in the N basin may be further strengthened by sparse vegetation, particularly in the deeper parts of the Mined habitat, as stickleback cannot hide as easily from predators (Ólafsdóttir et al. 2007a; Ólafsdóttir and Snorrason 2009).

We found subtle differences among habitats (but not between basins) in feeding structures: stickleback from the Mined habitat had wider gill raker gaps than stickleback from other habitats, and stickleback from the Warm habitat tended to have more gill rakers than stickleback from the Shore habitat. Such variation in feeding structures can reflect (plastic or genetic) responses to differences among habitats in prey type (e.g., McPhail 1994; Robinson and Wilson 1994; Smith and Skúlason 1996). Typically, longer and more numerous gill rakers, and narrower gaps, allow for better filtering of the water column and therefore increase the efficiency of catching zooplankton (Lavin and McPhail 1985; Robinson and Wilson 1994). In contrast to the “typical” divergence along the benthic–limnetic axis in stickleback (e.g., Lavin and McPhail 1985; McPhail 1994; McKinnon and Rundle 2002; Matthews et al. 2010), Mývatn stickleback population seems to consist of a broadly benthic feeder type as gill number ranged from 16 to 23 (Table 2), whereas that of typical benthic feeders may range from 15 to 21 and that of typical limnetic feeders from 22 to 28 (e.g., Kraak et al. 2001; Matthews et al. 2010).

Preliminary data on diet of the stickleback used in this study (A. Millet, B. K. Kristjánsson, Á. Einarsson and K. Räsänen, unpubl. data), as well as past diet data indicates that stickleback diet varies among habitats (Gudmundsson 1996; Kristjánsson et al. 2002; Koopmans 2010) and over time (Gudmundsson 1996). In June 2009, the frequency of stickleback primarily feeding on chironomid midges was higher in the Mined than in the Warm and Cladophorales habitats (proportion of individuals with at least 50% of chironomids as prey items: 71.6% vs. 41.7% vs. 24.1%, respectively; A. Millet, B. K. Kristjánsson, Á. Einarsson and K. Räsänen, unpubl. data), consistent with the relatively wider gill raker gaps of Mined stickleback. The apparent lack of divergence on a benthic–limnetic axis (i.e., many and long vs. few and short gill rakers) is also consistent with the fact that the alternative prey to midges in the shallow Lake Mývatn are benthic microcrustaceans rather than limnetic zooplankton (e.g., *Daphnia*) (Gudmundsson 1996). Prey-mediated selection may, hence, act to a different dimension or on different traits (e.g., feeding kinematics, McGee and Wainwright 2013) in Lake Mývatn than in deeper lakes.

Given the strong temporal variability in midge densities and stickleback diet (in low midge years, stickleback diet is dominated by benthic cladocerans and in high midge years by chironomid midge larvae; Gudmundsson 1996) an intriguing question is how such spatiotemporal dynamics affect diversification (e.g., Schluter 2000; Pelletier et al. 2009 and references therein; Scheiner and Holt 2012). The current evidence suggests (at least some) spatial divergence despite high temporal variability, and assessing the relative contribution of spatial and temporal variation to diversification of Lake Mývatn stickleback is part of ongoing work (A. Millet, B. K. Kristjánsson, Á. Einarsson and K. Räsänen, unpubl. data).

### Genetic structure

We found low neutral genetic structure and a lack of IBD across the lake, indicating extensive gene flow. However, Mined stickleback did differ subtly but significantly from Pondweed, Cladophorales, and Shore stickleback when all 12 loci where pooled. Pondweed stickleback differed in Gac1125 (locus linked to plate width; Colosimo et al. 2004) from Cladophorales stickleback and Warm stickleback in Stn26 (locus linked to spine length; Peichel et al. 2001; Mäkinen et al. 2008) from both Pondweed and Cladophorales stickleback. Plate number did not differ among habitats, but we currently lack data on plate width and hence we cannot associate Gac1125 to phenotypic variation. With regard to Stn26, the genetic divergence does not match the phenotypic pattern of defensive structure: we found no significant differences in spine length between Warm, Pondweed, and Cladophorales stickleback. This mismatch between genetic and phenotypic divergence could be due to a weak link between Stn26 and the gene associated to spine length in this stickleback population (referred to as “loose linkage”; Nosil et al. 2009). It could also result from lack of allelic variation in the gene under selection, which is common in European stickleback (Jones et al. 2012), with which Stn26 may be in close linkage disequilibrium. These results indicate at least some constraint to gene flow across the lake, but jointly with the subtle phenotypic divergence, also that divergent selection across the habitat types may be weak and/or that gene flow constrains adaptive divergence (e.g., Lenormand 2002; Garant et al. 2007; Räsänen and Hendry 2008), and/or that the young age (ca. 2300 generations, and less than 50 generations for the Mined habitat; Einarsson and Gulati 2004) of the Mývatn population renders divergence at the genomic level hard to detect with methods used here.

Previous work indicated the presence of two stickleback morphs in Lake Mývatn: the mud (equivalent to Pondweed habitat here) and lava (equivalent to Warm habitat here) morph, which differed in body size, head size, brain size, as well as in microsatellite and mtDNA markers (Kristjánsson et al. 2002; Ólafsdóttir et al. 2007b; Kotrschal et al. 2012). The phenotypic differences found in the present study are relatively consistent in that Warm stickleback differed in feeding morphology (GW and GRN) from Pondweed stickleback (Fig. 3). However, genetic divergence between Warm and Pondweed both for neutral (*F*_ST_ = 0.005) and putative QTL markers (max. *F*_ST_ = 0.032 for Stn26, Table 3) is much weaker than that found by Ólafsdóttir et al. (2007b) for microsatellites (*F*_ST_ = 0.082) or mtDNA (*F*_ST_ = 0.223). The reasons for this discrepancy are unclear, but include potential temporal variation in genetic structure (e.g., Crispo and Chapman 2010) and differences in number and type of microsatellites markers that were used (Here: 12 loci with six putative QTLs; Ólafsdóttir et al. 2007b: seven loci with four putative QTLs). With regard to the former, almost a decade separates the sampling periods and as this stickleback population undergoes strong interannual variability in environmental conditions and population densities (Ólafsson 1979; Einarsson and Gulati 2004) it is possible that environmental/population density changes have influenced genetic structure (e.g., Crispo and Chapman 2010).

Long-term monitoring of changes in extent of phenotypic divergence over time, coupled with temporally replicated studies on genetic structure, are needed to assess the potential impact of the environmental fluctuations on diversification of Mývatn stickleback and are part of our ongoing work.

### Selection or plasticity?

The lack of barriers to gene flow as well as the strong fluctuations in stickleback prey densities and type, combined with drastic fluctuations in stickleback density in Lake Mývatn (Einarsson and Gulati 2004), should favor phenotypic plasticity (Svanbäck et al. 2009; Pigliucci 2010) or increased trait variance (Bolnick 2011). We cannot currently distinguish whether the observed patterns reflect mostly phenotypic plasticity or genetically based variation as we used individuals collected from the wild. Both GRL and GW are often partially plastic in stickleback (e.g., Day et al. 1994; Berner et al. 2008), whereas GRN and defense traits are often strongly heritable (e.g., Lavin and McPhail 1985; McPhail 1994; Bittner et al. 2010; Loehr et al. 2012). Nevertheless, our results suggest that, despite extensive gene flow, the ecological differences among habitat types (or between basins) are strong enough to either promote plastic or genetic phenotypic divergence (e.g., West-Eberhard 2005) or phenotype-specific habitat sorting (e.g., Holt and Barfield 2008). Future research should determine the relative role of plastic versus genetic contributions to phenotypic variation in this system.

## Conclusions

In conclusion, we found subtle phenotypic and genetic divergence in Lake Mývatn stickleback, likely reflecting a combination of (genetic or plastic) responses to environmental differences, high gene flow and high temporal variability. The main differences were seen between Warm, Mined, and South basin habitats and reflected variation in defense (spine length) and feeding (GRN and GW) morphology, likely reflecting spatial variation in densities of gape-limited predators and prey. Future studies should assess the stability of such fine scale phenotypic divergence, particularly in connection with the strong fluctuations in ecological factors as typical in Lake Mývatn. Such spatiotemporal studies may give insight in processes influencing diversification of natural populations.

## Supporting Information

**Table S1.** Descriptive information of the 11 sampling sites (Fig. 1) in Lake Mývatn. The sites were divided in two basins and five habitat types. Abiotic parameters used to characterize habitats included water depth (m), average summer temperature (°C)^1^, dominant bottom substrate^2^, and biotic parameters included dominant species of Cladocera (%)^3^ and stickleback density (i.e., the total number of sticklebacks trapped). NA, not available.

**Figure S1.** Size frequency distribution of stickleback of threespine stickleback trapped in the North (A) and South (B) basin of Lake Mývatn. Only individuals larger than 40 mm were used (i.e., corresponding to adult population of stickleback).

**Figure S2.** LOSITAN output from the neutrality test of the 12 microsatellite markers used in the Mývatn stickleback population.
