# Supplementary material for: Spatial phenotypic and genetic structure of threespine stickleback (Gasterosteus aculeatus) in a heterogeneous natural system, Lake Mývatn, Iceland
Source: Ecol Evol. 2013 Aug 6;3(10):3219–32. doi: 10.1002/ece3.712 (PMC3797472; doi:10.1002/ece3.712)
Supplement: Supplementary file 1 [file ece30003-3219-SD1.docx]

| Table S1. Descriptive information of the 11 sampling sites (Figure 1) in Lake Mývatn. The sites were divided in two basins and five habitat types. Abiotic parameters used to characterize habitats included water depth (m), average summer temperature (°C)^1^, dominant bottom substrate^2^, and biotic parameters included dominant species of Cladocera (%)^3^ and stickleback density (i.e. the total number of sticklebacks trapped). NA: not available. | | | | | | | |
| --- | --- | --- | --- | --- | --- | --- | --- |
| *Basin* | *Habitat* | *Site* | *Depth* | *Temperature* | *Main substrate* | *Dominant Cladocera* | *Density* |
| North basin | Warm | HS1 | 0.10 | 21.1±3.6 | Lava strips covered by silica mud. Pondweed. | NA | 1952 |
|  |  | HS2 | 0.15 | 23.4± 0.8 | Lava strips covered by silica mud. Pondweed. | NA | 650 |
|  | Mined | 124 | 4.75 | 12.6±1.4 | Muddy bottom, no vegetation, anaerobic. | *Chydorus sphaericus* (86.0%) | 1836 |
|  | Pondweed | 128 | 1.62 | 13.0±1.7 | Pondweed, watermilfoil. | *Eurycercus lamellatus* (98.1%) | 1624 |
|  |  | DN | 1.18 | 13.0±2.6 | Pondweed, watermilfoil. | *Alona quadrangularis* (44.2%) | 1495 |
| South basin |  | 135 | 2.00 | 11.6±1.3 | Pondweed | *C. sphaericus* (35.7%) | 64 |
|  | Cladophorales | 41 | 3.03 | 12.1±1.4 | Cladophorales | *A. quadrangularis* (52.8%) | 36 |
|  |  | 44 | 2.95 | NA | Cladophorales | *E. lamellatus* (55.5%) | 147 |
|  |  | 23 | 3.24 | 12.2±1.6 | Cladophorales | *E. lamellatus* (52.0%) | 300 |
|  |  | 27 | 2.52 | 11.9±1.8 | Cladophorales | *E. lamellatus* (46.4%) | 32 |
|  | Shore | CS | 1.30 | 12.7±2.2 | Sparse rocks, sparse watermilfoil. | NA | 65 |
| ^1^Temperature average between 30 June 2011 and 18 August 2011 taken with a temperature logger (iButton Maxim Integrated Products, San Jose, CA, USA) placed at mid-depth and recording samples at three hours intervals; ^2^From Einarsson & Gulati (2004); ^3^Species composition (%) of benthic Cladocera (Einarsson & Gulati 2004). | | | | | | | |



¨





**Fig. S1.** Frequency distribution of stickleback of threespine stickleback trapped in the North (**a**) and South (**b**) basin of Lake Mývatn. Only individuals larger than 40 mm were used (i.e. corresponding to adult population of stickleback).


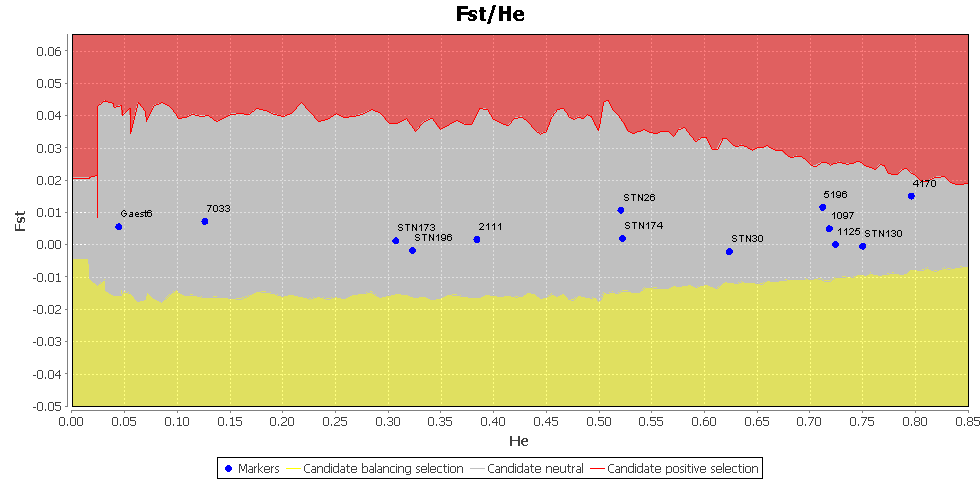


**Fig. S2**. LOSITAN output from the neutrality test of the 12 microsatellite markers used in the Mývatn stickleback population.
